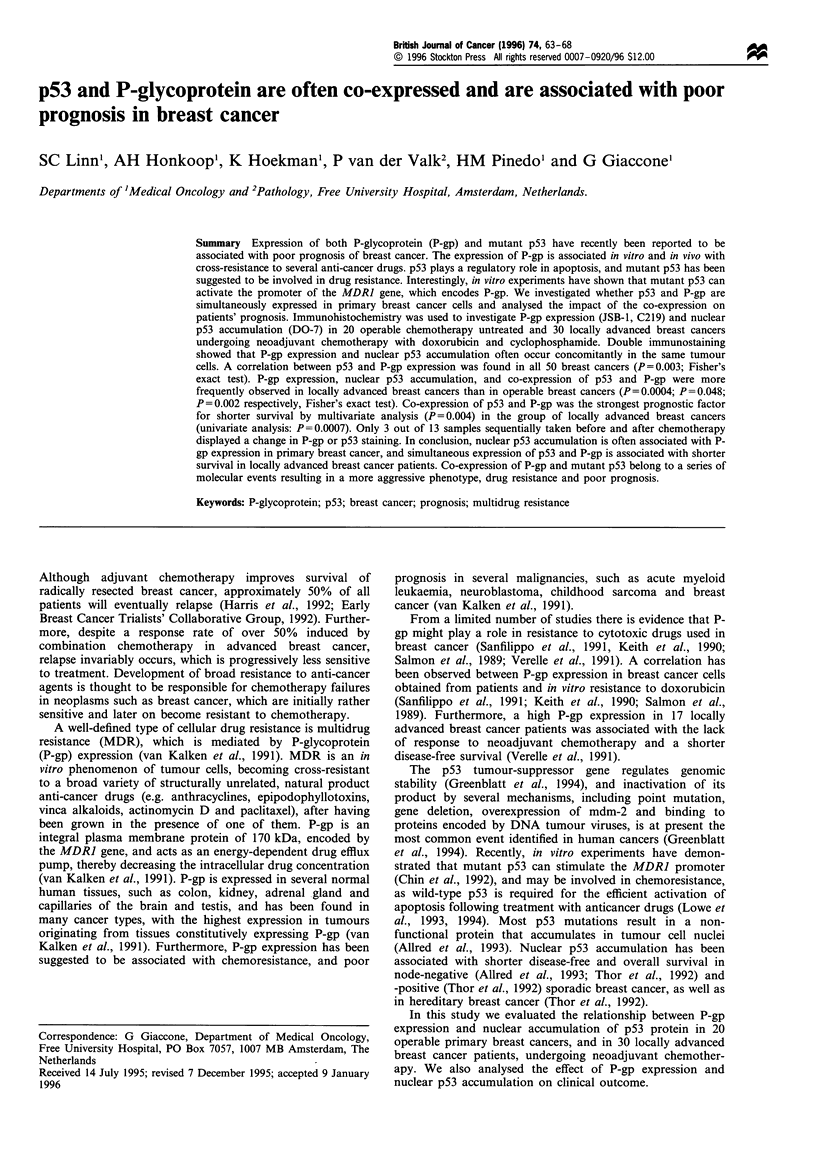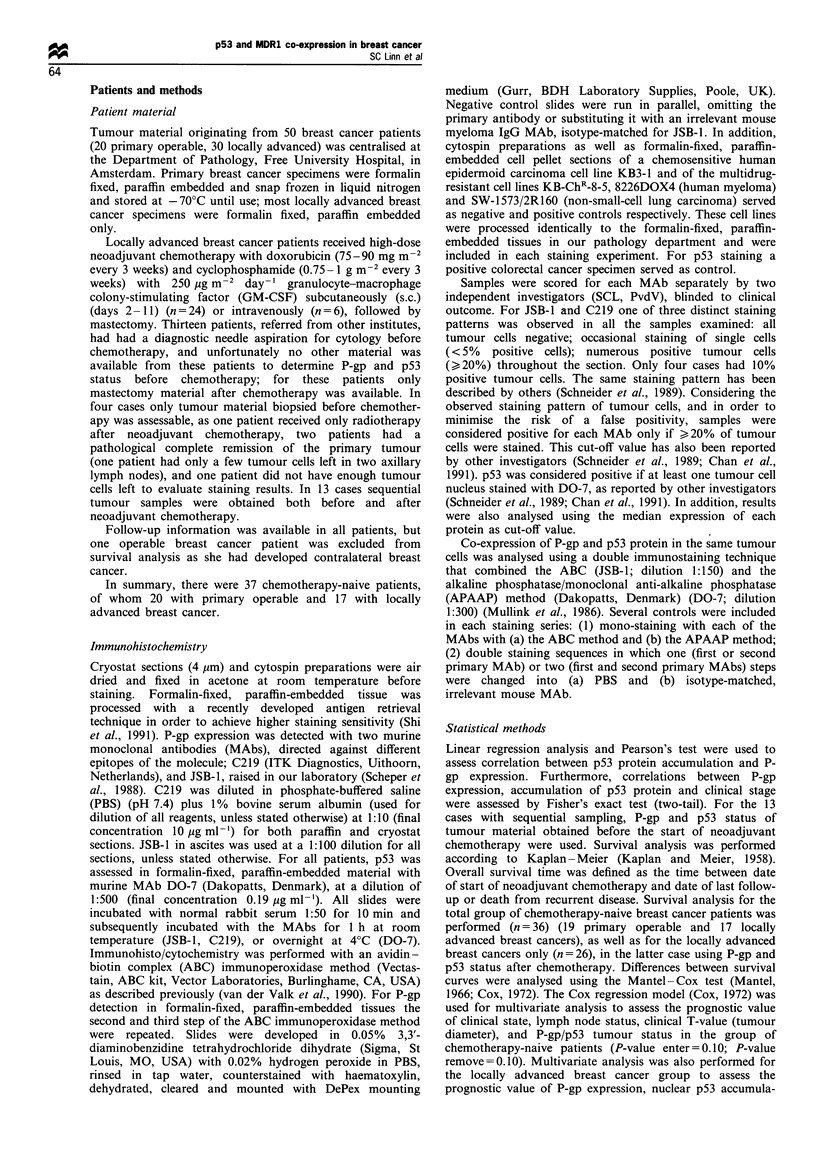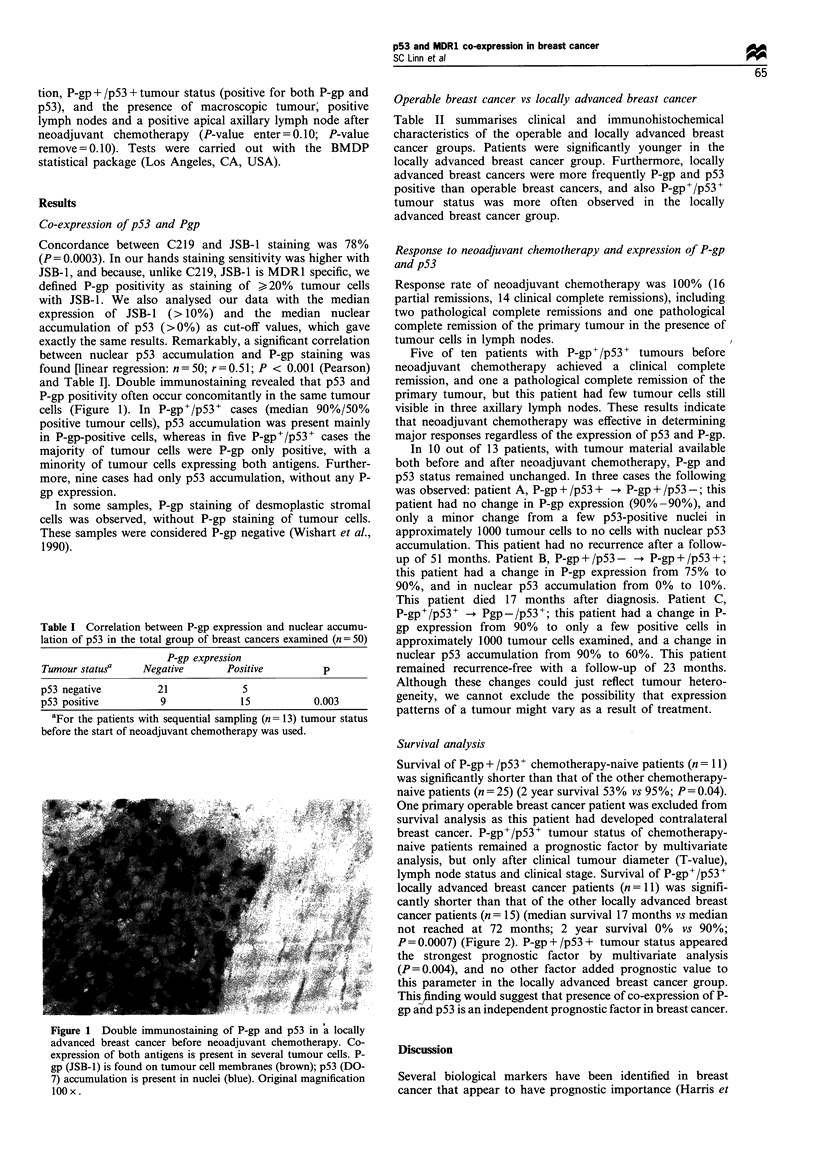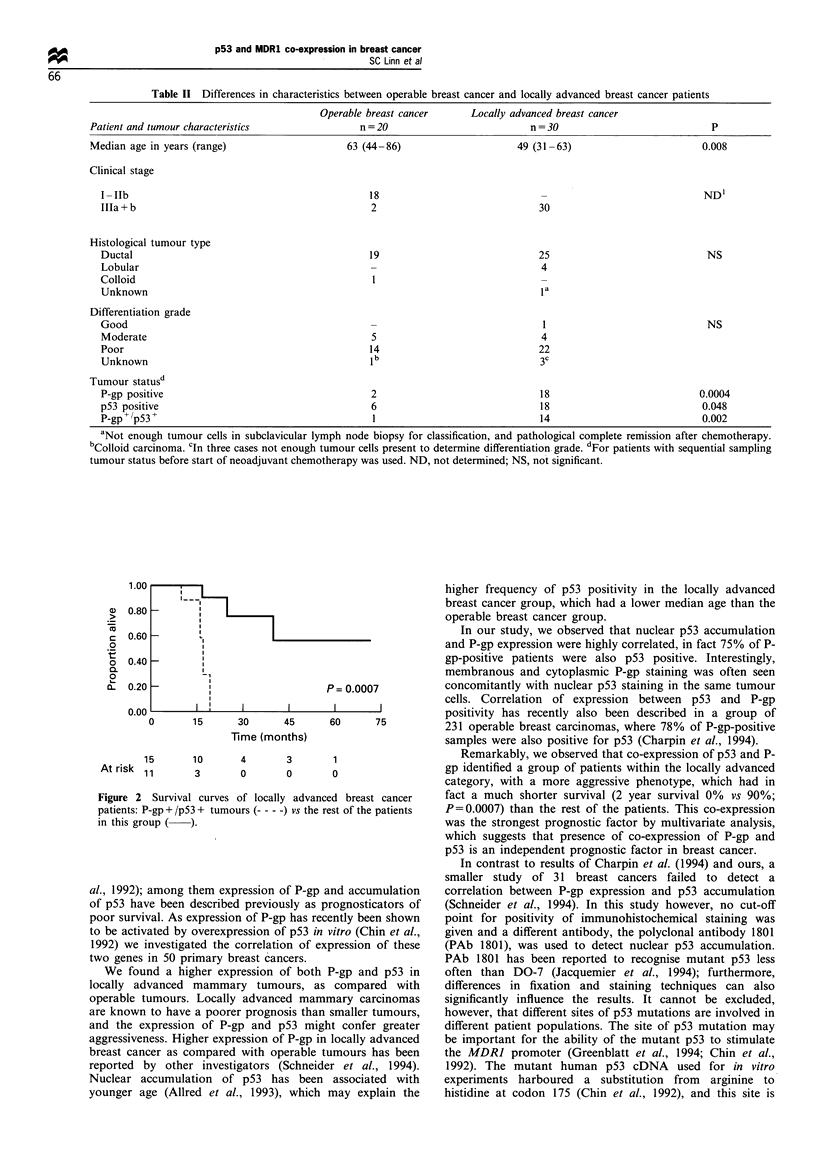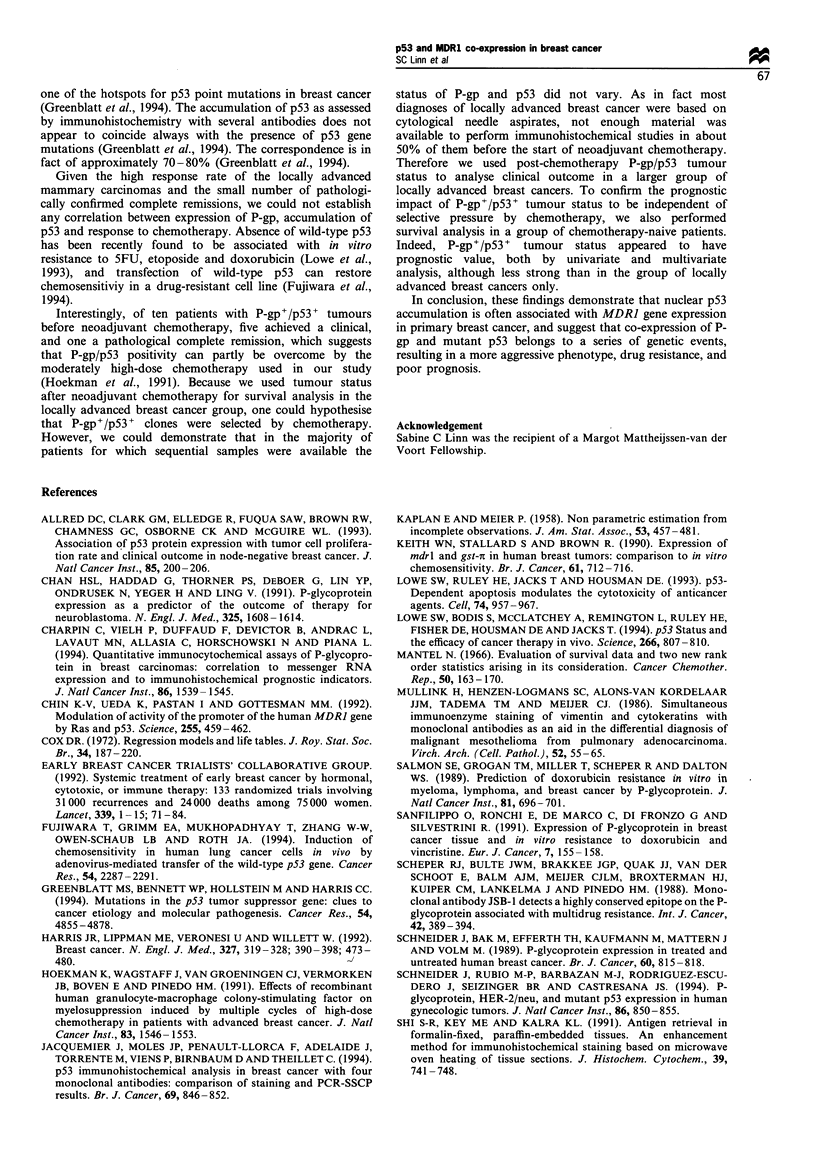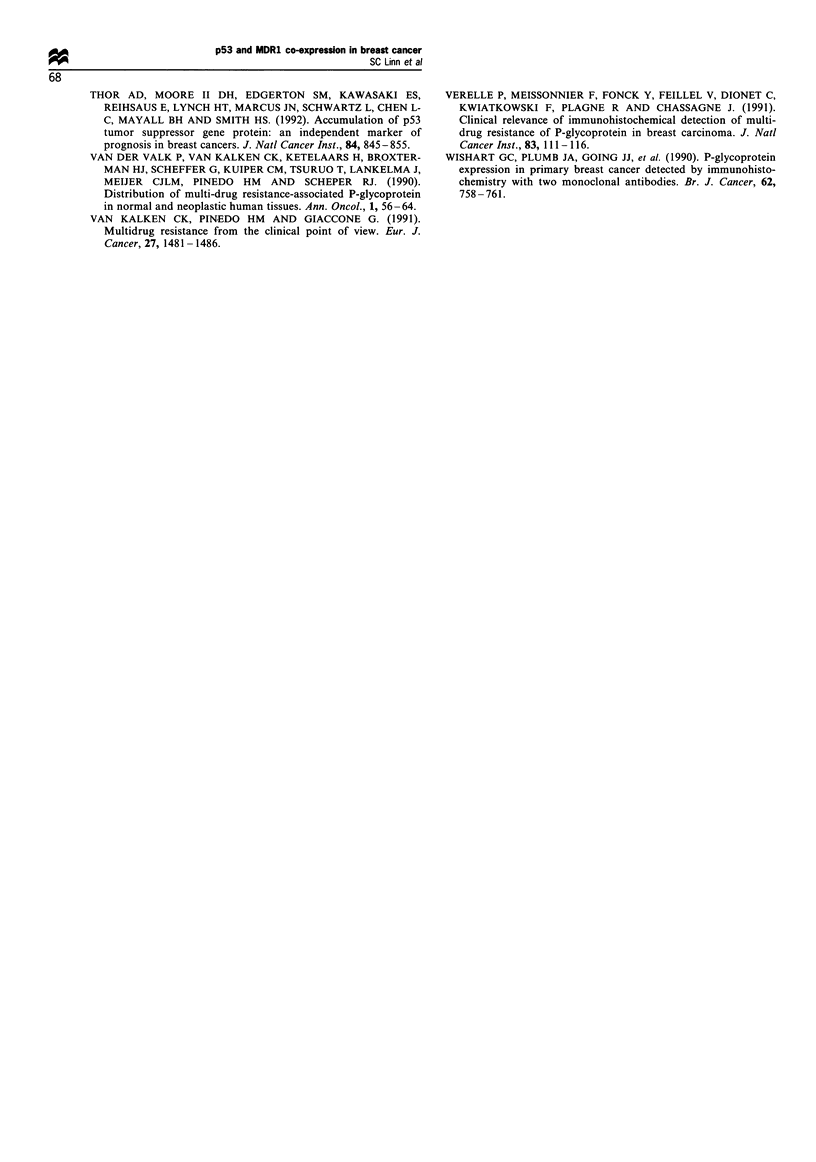# p53 and P-glycoprotein are often co-expressed and are associated with poor prognosis in breast cancer.

**DOI:** 10.1038/bjc.1996.316

**Published:** 1996-07

**Authors:** S. C. Linn, A. H. Honkoop, K. Hoekman, P. van der Valk, H. M. Pinedo, G. Giaccone

**Affiliations:** Department of Medical Oncology, Free University Hospital, Amsterdam, Netherlands.

## Abstract

**Images:**


British Journal of Cancer (1996) 74, 63-68
© 1996 Stockton Press All rights reserved 0007-0920/96 $12.00

### p53 and P-glycoprotein are often co-expressed and are associated with poor prognosis in breast cancer

SC Linn[1], AH Honkoop[1], K Hoekman[1], P van der Valk[2], HM Pinedo[1] and G Giaccone[1]

*Departments of [1]Medical Oncology and [2]Pathology, Free University Hospital, Amsterdam, Netherlands.*

**Summary** Expression of both P-glycoprotein (P-gp) and mutant p53 have recently been reported to be associated with poor prognosis of breast cancer. The expression of P-gp is associated *in vitro* and *in vivo* with cross-resistance to several anti-cancer drugs. p53 plays a regulatory role in apoptosis, and mutant p53 has been suggested to be involved in drug resistance. Interestingly, *in vitro* experiments have shown that mutant p53 can activate the promoter of the *MDR1* gene, which encodes P-gp. We investigated whether p53 and P-gp are simultaneously expressed in primary breast cancer cells and analysed the impact of the co-expression on patients' prognosis. Immunohistochemistry was used to investigate P-gp expression (JSB-1, C219) and nuclear p53 accumulation (DO-7) in 20 operable chemotherapy untreated and 30 locally advanced breast cancers undergoing neoadjuvant chemotherapy with doxorubicin and cyclophosphamide. Double immunostaining showed that P-gp expression and nuclear p53 accumulation often occur concomitantly in the same tumour cells. A correlation between p53 and P-gp expression was found in all 50 breast cancers ($P=0.003$; Fisher's exact test). P-gp expression, nuclear p53 accumulation, and co-expression of p53 and P-gp were more frequently observed in locally advanced breast cancers than in operable breast cancers ($P=0.0004$; $P=0.048$; $P=0.002$ respectively, Fisher's exact test). Co-expression of p53 and P-gp was the strongest prognostic factor for shorter survival by multivariate analysis ($P=0.004$) in the group of locally advanced breast cancers (univariate analysis: $P=0.0007$). Only 3 out of 13 samples sequentially taken before and after chemotherapy displayed a change in P-gp or p53 staining. In conclusion, nuclear p53 accumulation is often associated with P-gp expression in primary breast cancer, and simultaneous expression of p53 and P-gp is associated with shorter survival in locally advanced breast cancer patients. Co-expression of P-gp and mutant p53 belong to a series of molecular events resulting in a more aggressive phenotype, drug resistance and poor prognosis.

**Keywords:** P-glycoprotein; p53; breast cancer; prognosis; multidrug resistance

Although adjuvant chemotherapy improves survival of radically resected breast cancer, approximately 50% of all patients will eventually relapse (Harris *et al.*, 1992; Early Breast Cancer Trialists' Collaborative Group, 1992). Furthermore, despite a response rate of over 50% induced by combination chemotherapy in advanced breast cancer, relapse invariably occurs, which is progressively less sensitive to treatment. Development of broad resistance to anti-cancer agents is thought to be responsible for chemotherapy failures in neoplasms such as breast cancer, which are initially rather sensitive and later on become resistant to chemotherapy.

A well-defined type of cellular drug resistance is multidrug resistance (MDR), which is mediated by P-glycoprotein (P-gp) expression (van Kalken *et al.*, 1991). MDR is an *in vitro* phenomenon of tumour cells, becoming cross-resistant to a broad variety of structurally unrelated, natural product anti-cancer drugs (e.g. anthracyclines, epipodophyllotoxins, vinca alkaloids, actinomycin D and paclitaxel), after having been grown in the presence of one of them. P-gp is an integral plasma membrane protein of 170 kDa, encoded by the *MDR1* gene, and acts as an energy-dependent drug efflux pump, thereby decreasing the intracellular drug concentration (van Kalken *et al.*, 1991). P-gp is expressed in several normal human tissues, such as colon, kidney, adrenal gland and capillaries of the brain and testis, and has been found in many cancer types, with the highest expression in tumours originating from tissues constitutively expressing P-gp (van Kalken *et al.*, 1991). Furthermore, P-gp expression has been suggested to be associated with chemoresistance, and poor prognosis in several malignancies, such as acute myeloid leukaemia, neuroblastoma, childhood sarcoma and breast cancer (van Kalken *et al.*, 1991).

From a limited number of studies there is evidence that P-gp might play a role in resistance to cytotoxic drugs used in breast cancer (Sanfilippo *et al.*, 1991, Keith *et al.*, 1990; Salmon *et al.*, 1989; Verelle *et al.*, 1991). A correlation has been observed between P-gp expression in breast cancer cells obtained from patients and *in vitro* resistance to doxorubicin (Sanfilippo *et al.*, 1991; Keith *et al.*, 1990; Salmon *et al.*, 1989). Furthermore, a high P-gp expression in 17 locally advanced breast cancer patients was associated with the lack of response to neoadjuvant chemotherapy and a shorter disease-free survival (Verelle *et al.*, 1991).

The p53 tumour-suppressor gene regulates genomic stability (Greenblatt *et al.*, 1994), and inactivation of its product by several mechanisms, including point mutation, gene deletion, overexpression of mdm-2 and binding to proteins encoded by DNA tumour viruses, is at present the most common event identified in human cancers (Greenblatt *et al.*, 1994). Recently, *in vitro* experiments have demonstrated that mutant p53 can stimulate the *MDR1* promoter (Chin *et al.*, 1992), and may be involved in chemoresistance, as wild-type p53 is required for the efficient activation of apoptosis following treatment with anticancer drugs (Lowe *et al.*, 1993, 1994). Most p53 mutations result in a non-functional protein that accumulates in tumour cell nuclei (Allred *et al.*, 1993). Nuclear p53 accumulation has been associated with shorter disease-free and overall survival in node-negative (Allred *et al.*, 1993; Thor *et al.*, 1992) and -positive (Thor *et al.*, 1992) sporadic breast cancer, as well as in hereditary breast cancer (Thor *et al.*, 1992).

In this study we evaluated the relationship between P-gp expression and nuclear accumulation of p53 protein in 20 operable primary breast cancers, and in 30 locally advanced breast cancer patients, undergoing neoadjuvant chemotherapy. We also analysed the effect of P-gp expression and nuclear p53 accumulation on clinical outcome.

Correspondence: G Giaccone, Department of Medical Oncology, Free University Hospital, PO Box 7057, 1007 MB Amsterdam, The Netherlands
Received 14 July 1995; revised 7 December 1995; accepted 9 January 1996

### Patients and methods

### Patient material

Tumour material originating from 50 breast cancer patients (20 primary operable, 30 locally advanced) was centralised at the Department of Pathology, Free University Hospital, in Amsterdam. Primary breast cancer specimens were formalin fixed, paraffin embedded and snap frozen in liquid nitrogen and stored at −70°C until use; most locally advanced breast cancer specimens were formalin fixed, paraffin embedded only.

Locally advanced breast cancer patients received high-dose neoadjuvant chemotherapy with doxorubicin (75–90 mg $m^{-2}$ every 3 weeks) and cyclophosphamide (0.75–1 g $m^{-2}$ every 3 weeks) with 250 $\mu$g $m^{-2}$ $day^{-1}$ granulocyte–macrophage colony-stimulating factor (GM-CSF) subcutaneously (s.c.) (days 2–11) ($n=24$) or intravenously ($n=6$), followed by mastectomy. Thirteen patients, referred from other institutes, had had a diagnostic needle aspiration for cytology before chemotherapy, and unfortunately no other material was available from these patients to determine P-gp and p53 status before chemotherapy; for these patients only mastectomy material after chemotherapy was available. In four cases only tumour material biopsied before chemotherapy was assessable, as one patient received only radiotherapy after neoadjuvant chemotherapy, two patients had a pathological complete remission of the primary tumour (one patient had only a few tumour cells left in two axillary lymph nodes), and one patient did not have enough tumour cells left to evaluate staining results. In 13 cases sequential tumour samples were obtained both before and after neoadjuvant chemotherapy.

Follow-up information was available in all patients, but one operable breast cancer patient was excluded from survival analysis as she had developed contralateral breast cancer.

In summary, there were 37 chemotherapy-naive patients, of whom 20 with primary operable and 17 with locally advanced breast cancer.

### Immunohistochemistry

Cryostat sections (4 $\mu$m) and cytospin preparations were air dried and fixed in acetone at room temperature before staining. Formalin-fixed, paraffin-embedded tissue was processed with a recently developed antigen retrieval technique in order to achieve higher staining sensitivity (Shi *et al.*, 1991). P-gp expression was detected with two murine monoclonal antibodies (MAbs), directed against different epitopes of the molecule; C219 (ITK Diagnostics, Uithoorn, Netherlands), and JSB-1, raised in our laboratory (Scheper *et al.*, 1988). C219 was diluted in phosphate-buffered saline (PBS) (pH 7.4) plus 1% bovine serum albumin (used for dilution of all reagents, unless stated otherwise) at 1:10 (final concentration 10 $\mu$g $ml^{-1}$) for both paraffin and cryostat sections. JSB-1 in ascites was used at a 1:100 dilution for all sections, unless stated otherwise. For all patients, p53 was assessed in formalin-fixed, paraffin-embedded material with murine MAb DO-7 (Dakopatts, Denmark), at a dilution of 1:500 (final concentration 0.19 $\mu$g $ml^{-1}$). All slides were incubated with normal rabbit serum 1:50 for 10 min and subsequently incubated with the MAbs for 1 h at room temperature (JSB-1, C219), or overnight at 4°C (DO-7). Immunohisto/cytochemistry was performed with an avidin–biotin complex (ABC) immunoperoxidase method (Vectastain, ABC kit, Vector Laboratories, Burlinghame, CA, USA) as described previously (van der Valk *et al.*, 1990). For P-gp detection in formalin-fixed, paraffin-embedded tissues the second and third step of the ABC immunoperoxidase method were repeated. Slides were developed in 0.05% 3,3′-diaminobenzidine tetrahydrochloride dihydrate (Sigma, St Louis, MO, USA) with 0.02% hydrogen peroxide in PBS, rinsed in tap water, counterstained with haematoxylin, dehydrated, cleared and mounted with DePex mounting medium (Gurr, BDH Laboratory Supplies, Poole, UK). Negative control slides were run in parallel, omitting the primary antibody or substituting it with an irrelevant mouse myeloma IgG MAb, isotype-matched for JSB-1. In addition, cytospin preparations as well as formalin-fixed, paraffin-embedded cell pellet sections of a chemosensitive human epidermoid carcinoma cell line KB3-1 and of the multidrug-resistant cell lines KB-$Ch^R$-8-5, 8226DOX4 (human myeloma) and SW-1573/2R160 (non-small-cell lung carcinoma) served as negative and positive controls respectively. These cell lines were processed identically to the formalin-fixed, paraffin-embedded tissues in our pathology department and were included in each staining experiment. For p53 staining a positive colorectal cancer specimen served as control.

Samples were scored for each MAb separately by two independent investigators (SCL, PvdV), blinded to clinical outcome. For JSB-1 and C219 one of three distinct staining patterns was observed in all the samples examined: all tumour cells negative; occasional staining of single cells (<5% positive cells); numerous positive tumour cells ($\geqslant$20%) throughout the section. Only four cases had 10% positive tumour cells. The same staining pattern has been described by others (Schneider *et al.*, 1989). Considering the observed staining pattern of tumour cells, and in order to minimise the risk of a false positivity, samples were considered positive for each MAb only if $\geqslant$20% of tumour cells were stained. This cut-off value has also been reported by other investigators (Schneider *et al.*, 1989; Chan *et al.*, 1991). p53 was considered positive if at least one tumour cell nucleus stained with DO-7, as reported by other investigators (Schneider *et al.*, 1989; Chan *et al.*, 1991). In addition, results were also analysed using the median expression of each protein as cut-off value.

Co-expression of P-gp and p53 protein in the same tumour cells was analysed using a double immunostaining technique that combined the ABC (JSB-1; dilution 1:150) and the alkaline phosphatase/monoclonal anti-alkaline phosphatase (APAAP) method (Dakopatts, Denmark) (DO-7; dilution 1:300) (Mullink *et al.*, 1986). Several controls were included in each staining series: (1) mono-staining with each of the MAbs with (a) the ABC method and (b) the APAAP method; (2) double staining sequences in which one (first or second primary MAb) or two (first and second primary MAbs) steps were changed into (a) PBS and (b) isotype-matched, irrelevant mouse MAb.

### Statistical methods

Linear regression analysis and Pearson's test were used to assess correlation between p53 protein accumulation and P-gp expression. Furthermore, correlations between P-gp expression, accumulation of p53 protein and clinical stage were assessed by Fisher's exact test (two-tail). For the 13 cases with sequential sampling, P-gp and p53 status of tumour material obtained before the start of neoadjuvant chemotherapy were used. Survival analysis was performed according to Kaplan–Meier (Kaplan and Meier, 1958). Overall survival time was defined as the time between date of start of neoadjuvant chemotherapy and date of last follow-up or death from recurrent disease. Survival analysis for the total group of chemotherapy-naive breast cancer patients was performed ($n=36$) (19 primary operable and 17 locally advanced breast cancers), as well as for the locally advanced breast cancers only ($n=26$), in the latter case using P-gp and p53 status after chemotherapy. Differences between survival curves were analysed using the Mantel–Cox test (Mantel, 1966; Cox, 1972). The Cox regression model (Cox, 1972) was used for multivariate analysis to assess the prognostic value of clinical state, lymph node status, clinical T-value (tumour diameter), and P-gp/p53 tumour status in the group of chemotherapy-naive patients ($P$-value enter = 0.10; $P$-value remove = 0.10). Multivariate analysis was also performed for the locally advanced breast cancer group to assess the prognostic value of P-gp expression, nuclear p53 accumulation, P-gp + /p53 + tumour status (positive for both P-gp and p53), and the presence of macroscopic tumour, positive lymph nodes and a positive apical axillary lymph node after neoadjuvant chemotherapy (*P*-value enter = 0.10; *P*-value remove = 0.10). Tests were carried out with the BMDP statistical package (Los Angeles, CA, USA).

### Results

### *Co-expression of p53 and Pgp*

Concordance between C219 and JSB-1 staining was 78% ($P$ = 0.0003). In our hands staining sensitivity was higher with JSB-1, and because, unlike C219, JSB-1 is MDR1 specific, we defined P-gp positivity as staining of ≥20% tumour cells with JSB-1. We also analysed our data with the median expression of JSB-1 (>10%) and the median nuclear accumulation of p53 (>0%) as cut-off values, which gave exactly the same results. Remarkably, a significant correlation between nuclear p53 accumulation and P-gp staining was found [linear regression: $n$ = 50; $r$ = 0.51; $P < 0.001$ (Pearson) and Table I]. Double immunostaining revealed that p53 and P-gp positivity often occur concomitantly in the same tumour cells (Figure 1). In P-gp$^+$/p53$^+$ cases (median 90%/50% positive tumour cells), p53 accumulation was present mainly in P-gp-positive cells, whereas in five P-gp$^+$/p53$^+$ cases the majority of tumour cells were P-gp only positive, with a minority of tumour cells expressing both antigens. Furthermore, nine cases had only p53 accumulation, without any P-gp expression.

In some samples, P-gp staining of desmoplastic stromal cells was observed, without P-gp staining of tumour cells. These samples were considered P-gp negative (Wishart *et al.*, 1990).

**Table I** Correlation between P-gp expression and nuclear accumulation of p53 in the total group of breast cancers examined ($n$ = 50)

| Tumour status$^a$ | P-gp expression Negative | Positive | P |
|---|---|---|---|
| p53 negative | 21 | 5 | |
| p53 positive | 9 | 15 | 0.003 |

$^a$For the patients with sequential sampling ($n$ = 13) tumour status before the start of neoadjuvant chemotherapy was used.

**Figure 1** Double immunostaining of P-gp and p53 in a locally advanced breast cancer before neoadjuvant chemotherapy. Co-expression of both antigens is present in several tumour cells. P-gp (JSB-1) is found on tumour cell membranes (brown); p53 (DO-7) accumulation is present in nuclei (blue). Original magnification 100 ×.

### *Operable breast cancer vs locally advanced breast cancer*

Table II summarises clinical and immunohistochemical characteristics of the operable and locally advanced breast cancer groups. Patients were significantly younger in the locally advanced breast cancer group. Furthermore, locally advanced breast cancers were more frequently P-gp and p53 positive than operable breast cancers, and also P-gp$^+$/p53$^+$ tumour status was more often observed in the locally advanced breast cancer group.

### *Response to neoadjuvant chemotherapy and expression of P-gp and p53*

Response rate of neoadjuvant chemotherapy was 100% (16 partial remissions, 14 clinical complete remissions), including two pathological complete remissions and one pathological complete remission of the primary tumour in the presence of tumour cells in lymph nodes.

Five of ten patients with P-gp$^+$/p53$^+$ tumours before neoadjuvant chemotherapy achieved a clinical complete remission, and one a pathological complete remission of the primary tumour, but this patient had few tumour cells still visible in three axillary lymph nodes. These results indicate that neoadjuvant chemotherapy was effective in determining major responses regardless of the expression of p53 and P-gp.

In 10 out of 13 patients, with tumour material available both before and after neoadjuvant chemotherapy, P-gp and p53 status remained unchanged. In three cases the following was observed: patient A, P-gp + /p53 + → P-gp + /p53 −; this patient had no change in P-gp expression (90% − 90%), and only a minor change from a few p53-positive nuclei in approximately 1000 tumour cells to no cells with nuclear p53 accumulation. This patient had no recurrence after a follow-up of 51 months. Patient B, P-gp + /p53 − → P-gp + /p53 +; this patient had a change in P-gp expression from 75% to 90%, and in nuclear p53 accumulation from 0% to 10%. This patient died 17 months after diagnosis. Patient C, P-gp$^+$/p53$^+$ → Pgp − /p53$^+$; this patient had a change in P-gp expression from 90% to only a few positive cells in approximately 1000 tumour cells examined, and a change in nuclear p53 accumulation from 90% to 60%. This patient remained recurrence-free with a follow-up of 23 months. Although these changes could just reflect tumour heterogeneity, we cannot exclude the possibility that expression patterns of a tumour might vary as a result of treatment.

### *Survival analysis*

Survival of P-gp + /p53$^+$ chemotherapy-naive patients ($n$ = 11) was significantly shorter than that of the other chemotherapy-naive patients ($n$ = 25) (2 year survival 53% *vs* 95%; $P$ = 0.04). One primary operable breast cancer patient was excluded from survival analysis as this patient had developed contralateral breast cancer. P-gp$^+$/p53$^+$ tumour status of chemotherapy-naive patients remained a prognostic factor by multivariate analysis, but only after clinical tumour diameter (T-value), lymph node status and clinical stage. Survival of P-gp$^+$/p53$^+$ locally advanced breast cancer patients ($n$ = 11) was significantly shorter than that of the other locally advanced breast cancer patients ($n$ = 15) (median survival 17 months *vs* median not reached at 72 months; 2 year survival 0% *vs* 90%; $P$ = 0.0007) (Figure 2). P-gp + /p53 + tumour status appeared the strongest prognostic factor by multivariate analysis ($P$ = 0.004), and no other factor added prognostic value to this parameter in the locally advanced breast cancer group. This finding would suggest that presence of co-expression of P-gp and p53 is an independent prognostic factor in breast cancer.

### Discussion

Several biological markers have been identified in breast cancer that appear to have prognostic importance (Harris *et*

**Table II** Differences in characteristics between operable breast cancer and locally advanced breast cancer patients

| Patient and tumour characteristics | *Operable breast cancer* n=*20* | *Locally advanced breast cancer* n=*30* | P |
|---|---|---|---|
| Median age in years (range) | 63 (44–86) | 49 (31–63) | 0.008 |
| Clinical stage | | | |
| I–IIb | 18 | – | ND[1] |
| IIIa+b | 2 | 30 | |
| Histological tumour type | | | |
| Ductal | 19 | 25 | NS |
| Lobular | – | 4 | |
| Colloid | 1 | – | |
| Unknown | | 1[a] | |
| Differentiation grade | | | |
| Good | – | 1 | NS |
| Moderate | 5 | 4 | |
| Poor | 14 | 22 | |
| Unknown | 1[b] | 3[c] | |
| Tumour status[d] | | | |
| P-gp positive | 2 | 18 | 0.0004 |
| p53 positive | 6 | 18 | 0.048 |
| P-gp$^+$/p53$^+$ | 1 | 14 | 0.002 |

[a]Not enough tumour cells in subclavicular lymph node biopsy for classification, and pathological complete remission after chemotherapy. [b]Colloid carcinoma. [c]In three cases not enough tumour cells present to determine differentiation grade. [d]For patients with sequential sampling tumour status before start of neoadjuvant chemotherapy was used. ND, not determined; NS, not significant.

**Figure 2** Survival curves of locally advanced breast cancer patients: P-gp+/p53+ tumours (- - - -) *vs* the rest of the patients in this group (——).

*al.*, 1992); among them expression of P-gp and accumulation of p53 have been described previously as prognosticators of poor survival. As expression of P-gp has recently been shown to be activated by overexpression of p53 *in vitro* (Chin *et al.*, 1992) we investigated the correlation of expression of these two genes in 50 primary breast cancers.

We found a higher expression of both P-gp and p53 in locally advanced mammary tumours, as compared with operable tumours. Locally advanced mammary carcinomas are known to have a poorer prognosis than smaller tumours, and the expression of P-gp and p53 might confer greater aggressiveness. Higher expression of P-gp in locally advanced breast cancer as compared with operable tumours has been reported by other investigators (Schneider *et al.*, 1994). Nuclear accumulation of p53 has been associated with younger age (Allred *et al.*, 1993), which may explain the higher frequency of p53 positivity in the locally advanced breast cancer group, which had a lower median age than the operable breast cancer group.

In our study, we observed that nuclear p53 accumulation and P-gp expression were highly correlated, in fact 75% of P-gp-positive patients were also p53 positive. Interestingly, membranous and cytoplasmic P-gp staining was often seen concomitantly with nuclear p53 staining in the same tumour cells. Correlation of expression between p53 and P-gp positivity has recently also been described in a group of 231 operable breast carcinomas, where 78% of P-gp-positive samples were also positive for p53 (Charpin *et al.*, 1994).

Remarkably, we observed that co-expression of p53 and P-gp identified a group of patients within the locally advanced category, with a more aggressive phenotype, which had in fact a much shorter survival (2 year survival 0% *vs* 90%; $P=0.0007$) than the rest of the patients. This co-expression was the strongest prognostic factor by multivariate analysis, which suggests that presence of co-expression of P-gp and p53 is an independent prognostic factor in breast cancer.

In contrast to results of Charpin *et al.* (1994) and ours, a smaller study of 31 breast cancers failed to detect a correlation between P-gp expression and p53 accumulation (Schneider *et al.*, 1994). In this study however, no cut-off point for positivity of immunohistochemical staining was given and a different antibody, the polyclonal antibody 1801 (PAb 1801), was used to detect nuclear p53 accumulation. PAb 1801 has been reported to recognise mutant p53 less often than DO-7 (Jacquemier *et al.*, 1994); furthermore, differences in fixation and staining techniques can also significantly influence the results. It cannot be excluded, however, that different sites of p53 mutations are involved in different patient populations. The site of p53 mutation may be important for the ability of the mutant p53 to stimulate the *MDR1* promoter (Greenblatt *et al.*, 1994; Chin *et al.*, 1992). The mutant human p53 cDNA used for *in vitro* experiments harboured a substitution from arginine to histidine at codon 175 (Chin *et al.*, 1992), and this site is one of the hotspots for p53 point mutations in breast cancer (Greenblatt *et al.*, 1994). The accumulation of p53 as assessed by immunohistochemistry with several antibodies does not appear to coincide always with the presence of p53 gene mutations (Greenblatt *et al.*, 1994). The correspondence is in fact of approximately 70–80% (Greenblatt *et al.*, 1994).

Given the high response rate of the locally advanced mammary carcinomas and the small number of pathologically confirmed complete remissions, we could not establish any correlation between expression of P-gp, accumulation of p53 and response to chemotherapy. Absence of wild-type p53 has been recently found to be associated with *in vitro* resistance to 5FU, etoposide and doxorubicin (Lowe *et al.*, 1993), and transfection of wild-type p53 can restore chemosensitiviy in a drug-resistant cell line (Fujiwara *et al.*, 1994).

Interestingly, of ten patients with P-gp$^+$/p53$^+$ tumours before neoadjuvant chemotherapy, five achieved a clinical, and one a pathological complete remission, which suggests that P-gp/p53 positivity can partly be overcome by the moderately high-dose chemotherapy used in our study (Hoekman *et al.*, 1991). Because we used tumour status after neoadjuvant chemotherapy for survival analysis in the locally advanced breast cancer group, one could hypothesise that P-gp$^+$/p53$^+$ clones were selected by chemotherapy. However, we could demonstrate that in the majority of patients for which sequential samples were available the status of P-gp and p53 did not vary. As in fact most diagnoses of locally advanced breast cancer were based on cytological needle aspirates, not enough material was available to perform immunohistochemical studies in about 50% of them before the start of neoadjuvant chemotherapy. Therefore we used post-chemotherapy P-gp/p53 tumour status to analyse clinical outcome in a larger group of locally advanced breast cancers. To confirm the prognostic impact of P-gp$^+$/p53$^+$ tumour status to be independent of selective pressure by chemotherapy, we also performed survival analysis in a group of chemotherapy-naive patients. Indeed, P-gp$^+$/p53$^+$ tumour status appeared to have prognostic value, both by univariate and multivariate analysis, although less strong than in the group of locally advanced breast cancers only.

In conclusion, these findings demonstrate that nuclear p53 accumulation is often associated with *MDR1* gene expression in primary breast cancer, and suggest that co-expression of P-gp and mutant p53 belongs to a series of genetic events, resulting in a more aggressive phenotype, drug resistance, and poor prognosis.

**Acknowledgement**
Sabine C Linn was the recipient of a Margot Mattheijssen-van der Voort Fellowship.

### References

ALLRED DC, CLARK GM, ELLEDGE R, FUQUA SAW, BROWN RW, CHAMNESS GC, OSBORNE CK AND MCGUIRE WL. (1993). Association of p53 protein expression with tumor cell proliferation rate and clinical outcome in node-negative breast cancer. *J. Natl Cancer Inst.*, **85**, 200–206.

CHAN HSL, HADDAD G, THORNER PS, DEBOER G, LIN YP, ONDRUSEK N, YEGER H AND LING V. (1991). P-glycoprotein expression as a predictor of the outcome of therapy for neuroblastoma. *N. Engl. J. Med.*, **325**, 1608–1614.

CHARPIN C, VIELH P, DUFFAUD F, DEVICTOR B, ANDRAC L, LAVAUT MN, ALLASIA C, HORSCHOWSKI N AND PIANA L. (1994). Quantitative immunocytochemical assays of P-glycoprotein in breast carcinomas: correlation to messenger RNA expression and to immunohistochemical prognostic indicators. *J. Natl Cancer Inst.*, **86**, 1539–1545.

CHIN K-V, UEDA K, PASTAN I AND GOTTESMAN MM. (1992). Modulation of activity of the promoter of the human *MDR1* gene by Ras and p53. *Science*, **255**, 459–462.

COX DR. (1972). Regression models and life tables. *J. Roy. Stat. Soc. Br.*, **34**, 187–220.

EARLY BREAST CANCER TRIALISTS' COLLABORATIVE GROUP. (1992). Systemic treatment of early breast cancer by hormonal, cytotoxic, or immune therapy: 133 randomized trials involving 31 000 recurrences and 24 000 deaths among 75 000 women. *Lancet*, **339**, 1–15; 71–84.

FUJIWARA T, GRIMM EA, MUKHOPADHYAY T, ZHANG W-W, OWEN-SCHAUB LB AND ROTH JA. (1994). Induction of chemosensitivity in human lung cancer cells *in vivo* by adenovirus-mediated transfer of the wild-type *p53* gene. *Cancer Res.*, **54**, 2287–2291.

GREENBLATT MS, BENNETT WP, HOLLSTEIN M AND HARRIS CC. (1994). Mutations in the *p53* tumor suppressor gene: clues to cancer etiology and molecular pathogenesis. *Cancer Res.*, **54**, 4855–4878.

HARRIS JR, LIPPMAN ME, VERONESI U AND WILLETT W. (1992). Breast cancer. *N. Engl. J. Med.*, **327**, 319–328; 390–398; 473–480.

HOEKMAN K, WAGSTAFF J, VAN GROENINGEN CJ, VERMORKEN JB, BOVEN E AND PINEDO HM. (1991). Effects of recombinant human granulocyte-macrophage colony-stimulating factor on myelosuppression induced by multiple cycles of high-dose chemotherapy in patients with advanced breast cancer. *J. Natl Cancer Inst.*, **83**, 1546–1553.

JACQUEMIER J, MOLES JP, PENAULT-LLORCA F, ADELAIDE J, TORRENTE M, VIENS P, BIRNBAUM D AND THEILLET C. (1994). p53 immunohistochemical analysis in breast cancer with four monoclonal antibodies: comparison of staining and PCR-SSCP results. *Br. J. Cancer*, **69**, 846–852.

KAPLAN E AND MEIER P. (1958). Non parametric estimation from incomplete observations. *J. Am. Stat. Assoc.*, **53**, 457–481.

KEITH WN, STALLARD S AND BROWN R. (1990). Expression of *mdr*1 and *gst-π* in human breast tumors: comparison to *in vitro* chemosensitivity. *Br. J. Cancer*, **61**, 712–716.

LOWE SW, RULEY HE, JACKS T AND HOUSMAN DE. (1993). p53-Dependent apoptosis modulates the cytotoxicity of anticancer agents. *Cell*, **74**, 957–967.

LOWE SW, BODIS S, MCCLATCHEY A, REMINGTON L, RULEY HE, FISHER DE, HOUSMAN DE AND JACKS T. (1994). *p53* Status and the efficacy of cancer therapy in vivo. *Science*, **266**, 807–810.

MANTEL N. (1966). Evaluation of survival data and two new rank order statistics arising in its consideration. *Cancer Chemother. Rep.*, **50**, 163–170.

MULLINK H, HENZEN-LOGMANS SC, ALONS-VAN KORDELAAR JJM, TADEMA TM AND MEIJER CJ. (1986). Simultaneous immunoenzyme staining of vimentin and cytokeratins with monoclonal antibodies as an aid in the differential diagnosis of malignant mesothelioma from pulmonary adenocarcinoma. *Virch. Arch. (Cell. Pathol.)*, **52**, 55–65.

SALMON SE, GROGAN TM, MILLER T, SCHEPER R AND DALTON WS. (1989). Prediction of doxorubicin resistance *in vitro* in myeloma, lymphoma, and breast cancer by P-glycoprotein. *J. Natl Cancer Inst.*, **81**, 696–701.

SANFILIPPO O, RONCHI E, DE MARCO C, DI FRONZO G AND SILVESTRINI R. (1991). Expression of P-glycoprotein in breast cancer tissue and *in vitro* resistance to doxorubicin and vincristine. *Eur. J. Cancer*, **7**, 155–158.

SCHEPER RJ, BULTE JWM, BRAKKEE JGP, QUAK JJ, VAN DER SCHOOT E, BALM AJM, MEIJER CJLM, BROXTERMAN HJ, KUIPER CM, LANKELMA J AND PINEDO HM. (1988). Monoclonal antibody JSB-1 detects a highly conserved epitope on the P-glycoprotein associated with multidrug resistance. *Int. J. Cancer*, **42**, 389–394.

SCHNEIDER J, BAK M, EFFERTH TH, KAUFMANN M, MATTERN J AND VOLM M. (1989). P-glycoprotein expression in treated and untreated human breast cancer. *Br. J. Cancer*, **60**, 815–818.

SCHNEIDER J, RUBIO M-P, BARBAZAN M-J, RODRIGUEZ-ESCUDERO J, SEIZINGER BR AND CASTRESANA JS. (1994). P-glycoprotein, HER-2/neu, and mutant p53 expression in human gynecologic tumors. *J. Natl Cancer Inst.*, **86**, 850–855.

SHI S-R, KEY ME AND KALRA KL. (1991). Antigen retrieval in formalin-fixed, paraffin-embedded tissues. An enhancement method for immunohistochemical staining based on microwave oven heating of tissue sections. *J. Histochem. Cytochem.*, **39**, 741–748.

THOR AD, MOORE II DH, EDGERTON SM, KAWASAKI ES, REIHSAUS E, LYNCH HT, MARCUS JN, SCHWARTZ L, CHEN L-C, MAYALL BH AND SMITH HS. (1992). Accumulation of p53 tumor suppressor gene protein: an independent marker of prognosis in breast cancers. *J. Natl Cancer Inst.*, **84,** 845–855.

VAN DER VALK P, VAN KALKEN CK, KETELAARS H, BROXTERMAN HJ, SCHEFFER G, KUIPER CM, TSURUO T, LANKELMA J, MEIJER CJLM, PINEDO HM AND SCHEPER RJ. (1990). Distribution of multi-drug resistance-associated P-glycoprotein in normal and neoplastic human tissues. *Ann. Oncol.*, **1,** 56–64.

VAN KALKEN CK, PINEDO HM AND GIACCONE G. (1991). Multidrug resistance from the clinical point of view. *Eur. J. Cancer*, **27,** 1481–1486.

VERELLE P, MEISSONNIER F, FONCK Y, FEILLEL V, DIONET C, KWIATKOWSKI F, PLAGNE R AND CHASSAGNE J. (1991). Clinical relevance of immunohistochemical detection of multidrug resistance of P-glycoprotein in breast carcinoma. *J. Natl Cancer Inst.*, **83,** 111–116.

WISHART GC, PLUMB JA, GOING JJ, *et al.* (1990). P-glycoprotein expression in primary breast cancer detected by immunohistochemistry with two monoclonal antibodies. *Br. J. Cancer*, **62,** 758–761.